# Aluminum-Induced Entropy in Biological Systems: Implications for Neurological Disease

**DOI:** 10.1155/2014/491316

**Published:** 2014-10-02

**Authors:** Christopher A. Shaw, Stephanie Seneff, Stephen D. Kette, Lucija Tomljenovic, John W. Oller, Robert M. Davidson

**Affiliations:** ^1^Neural Dynamics Research Group, Department of Ophthalmology and Visual Sciences, 828 W. 10th Avenue, Vancouver, British Columbia, Canada V5Z 1L8; ^2^Program Experimental Medicine, University of British Columbia, Vancouver, Canada V5Z 1L8; ^3^Program in Neurosciences, University of British Columbia, Vancouver, Canada V5Z 1L8; ^4^MIT Computer Science and Artificial Intelligence Laboratory, 32 Vassar Street, Cambridge, MA 02139, USA; ^5^Hudson, FL 34667, USA; ^6^Department of Communicative Disorders, University of Louisiana, Lafayette, LA 70504-3170, USA; ^7^Internal Medicine Group Practice, PhyNet Inc., 4002 Technology Center, Longview, TX 75605, USA

## Abstract

Over the last 200 years, mining, smelting, and refining of aluminum (Al) in various forms have increasingly exposed living species to this naturally abundant metal. Because of its prevalence in the earth's crust, prior to its recent uses it was regarded as inert and therefore harmless. However, Al is invariably toxic to living systems and has no known beneficial role in any biological systems. Humans are increasingly exposed to Al from food, water, medicinals, vaccines, and cosmetics, as well as from industrial occupational exposure. Al disrupts biological self-ordering, energy transduction, and signaling systems, thus increasing biosemiotic entropy. Beginning with the biophysics of water, disruption progresses through the macromolecules that are crucial to living processes (DNAs, RNAs, proteoglycans, and proteins). It injures cells, circuits, and subsystems and can cause catastrophic failures ending in death. Al forms toxic complexes with other elements, such as fluorine, and interacts negatively with mercury, lead, and glyphosate. Al negatively impacts the central nervous system in all species that have been studied, including humans. Because of the global impacts of Al on water dynamics and biosemiotic systems, CNS disorders in humans are sensitive indicators of the Al toxicants to which we are being exposed.

## 1. Introduction

Aluminum (Al) is the most common metal and the third most abundant element in the earth's crust [1–3]. However, it seems to have no beneficial role in the biochemistry of any biota [1]. Until the 1820s when the industrial extraction of Al, primarily from bauxite ore [4], made it possible to bring Al into food processing, manufacturing, medicines, cosmetics, vaccines, and other applications, Al was almost completely absent from the biosphere [5]. Concerns about the toxicity of ingesting Al were expressed over 100 years ago [6]. Today, biologically ingested or injected forms include salts of Al in processed foods [7] and medicinal products [8] such as antacids, glossy coatings for pills, and vaccine* adjuvants*. The last use, which portrays Al compounds as “helpers”—the English translation of the Latin root of* adjuvants*—is supposed to shock the recipient's immune defenses into action, ostensibly to enhance the immunogenicity of the pathogen(s) in the vaccine(s) [9]. Al salts are also found in dyes [10], cosmetics [5], antiperspirants [11–14], sunscreens [15, 16], and thousands of material products including foils, food containers, and utensils.

In this paper, we will show that Al is harmful to the CNS, acting in a number of deleterious ways and across multiple levels, to induce biosemiotic entropy [17]. A countervailing view exists [18–20], but the assertions of safety are invariably based on weak epidemiological designs, ones that overwhelm significant negative signals with irrelevant noise factors. Such studies that fail to detect significant negative outcomes neither stand up to rigorous scrutiny nor outweigh better designed research, in a vast and growing literature, showing significant negative impacts sustaining the central hypothesis of this paper. Irrefutable research evidence shows that Al exposure is harmful. Further, results discussed in this paper show that it is counterfactual for researchers to argue that Al is universally safe or beneficial even in trace amounts.

Al is used extensively in food processing, for example, in Al-mordanted dye lakes for food coloring, in coatings for pharmaceutical tablets and vitamin capsules, for emulsifying, as a rising agent, to thicken gravies, and in meat-binders, stabilizing agents and texturizers [18]. Even drinking water is a source of Al exposure, although the amount contained in drinking water is typically far below concentrations in common antacids [21]. However, there is concern that the Al in drinking water may be more easily absorbed than at mealtime, due to the fact that an empty stomach promotes absorption [21]. Alum (Al sulfate or Al potassium sulfate) is commonly used in water treatment plants as a coagulant to allow negatively charged colloidal particles to clump together for easy removal. Epidemiological studies have shown that people living in districts with higher Al burden in drinking water are more likely to be diagnosed with Alzheimer's disease [22].

Because tea plants contain a higher concentration of Al than many other plants, and, because tea beverages are consumed in large quantities worldwide, a high incidence of Al exposure comes through drinking tea [23]. Al content in tea ranges from 2 to 6 mg/L [24]. Tea infusions have been analyzed for the speciation of Al content, and it has been determined that it is typically bound to large organic molecules such as polyphenols or to citrate [24, 25]. Tea typically contains much more Al than water, and so tea becomes a significant source of Al for heavy tea drinkers. An experiment to estimate oral Al bioavailability from tea involving 8 rats was conducted by injecting Al citrate into tea leaves, delivering approximately the same amount of Al as is inherently found in tea leaves (0.5 to 1 mg/gm) [26]. The brewed tea was administered through intragastric infusion. Following infusion, peak serum levels of Al were up to 1500-fold above mean pretreatment values.

In a substantial and recent review of research, Walton [27] concludes that Alzheimer's disease is a manifestation of chronic Al neurotoxicity in humans. Because Al is similar to iron, it gains access to iron-dependent cells involved in memory. As it accumulates over time in such cells, it causes microtubule depletion and disables neuronal afferents and efferents resulting in the multiregion atrophy characteristic of Alzheimer's pathology [27].  Table 1 highlights some of the Al compounds to which humans are commonly exposed which are known to have deleterious effects on the central nervous systems (CNS) of both animals and humans [28], whereas Tables 2 and 3, respectively, present Al intake data, and its physical properties compared to other metals.  Table 1 also shows dosage and known effects of each source on animals and/or humans.

Al in all of the forms studied, as Table 1 shows, produces harmful effects in living organisms: it especially harms the CNS. In studies involving* in vitro* cultures of neuronal-glial cells, the ROS-generating capabilities of several physiologically relevant neurotoxic factors were compared [29, 30]. It was found that Al-sulfate was the most potent single metal sulfate inducer of ROS, as well as the most potent combinatorial inducer in conjunction with Fe. Nanomolar concentrations of Al were sufficient to induce ROS and proinflammatory gene expression. Nanomolar concentrations of Al-sulfate upregulated the expression of several genes implicated in Alzheimer's disease, including proinflammatory and proapoptotic gene expression [30].

Given the fact that there are no known biochemical reactions that require Al, should it be surprising that introducing it into living organisms commonly leads to pathological outcomes [31–46]? Because of its +3 charge, Al attracts negatively charged ions and electrons, but because it cannot transition to other oxidation states besides +3, it is not a component in any redox reactions. Oxygen, carbon, hydrogen, nitrogen, calcium, and phosphorous constitute 99% of human body mass, with the remaining 1% consisting of potassium, sulfur, sodium, chlorine, and magnesium, as well as trace elements such as fluorine, selenium, and zinc, and xenobiotic (biologically foreign and usually toxic) elements such as titanium, mercury, and lead [47]. Thus, Al can end up in many biochemical contexts in theory, but in fact some atoms and molecules are far more likely to react with Al compounds [48]. Among the most vulnerable molecules are those most directly involved in self-ordering, self-assembling systems of biosemiotics that work like multilayered, interrelated languages. The best known macromolecules that are susceptible to minute but often disabling injuries by Al compounds are DNA molecules that must be translated via the assistance of a growing multitude of RNA molecules into proteins. The latter in turn are essential to the structure and functions of the whole society of cells [49], tissues, and organ systems. Formerly, it was thought, following the Crick dogma [50], that communications were essentially a one-way street from DNA to RNA to protein, but it has more recently been argued [17, 51, 52] that communications involve more complex bidirectional interactions among those macromolecules, such that the genome is informed concerning what is going on in the environment. The dynamical matrix of negative charge densities in heparan sulfate proteoglycans (HSPGs), as modulated in time and space by interfacial water, exchanging between the first few solvation layers and bulk, might prove to be the supramolecular physical basis for* informing* the genome over distance [53].

There are estimated to be 20,000–25,000 protein coding genes in the human genome [54] and even more variant proteins possible through posttranslational modifications estimated to be upwards of 100,000. Thus there are many macromolecules with which Al^3+^ species can interact, either directly or indirectly. Eukaryotic proteins are polymers of various combinations and lengths consisting of an array of 23 amino acids joined by peptide bonds. Each of the 23 amino acids has a unique side chain consisting of various organic substituents. Al can interact with the side chains [55], some of which—serine, threonine, and tyrosine—are phosphorylated, enabling phosphoregulation of enzyme activity and binding with other proteins. Al can disrupt all of these side chains and the processes dependent on them [56]. Cysteine, methionine, homocysteine, and glutathione contain sulfur, and they are intermediaries instrumental in methylation and transsulfuration pathways, as well as in heavy metal detoxification. These processes can be disrupted by Al [57] because of the strong binding affinity of Al with sulfur oxyanions. Glutamic and aspartic acids have negatively charged carboxylate side chains. Al has a much stronger binding affinity to these side chains, for instance, than the nontoxic cation, magnesium [58].

Therefore, Al is ineffective in redox reactions, though its +3 charge makes it likely to adsorb to suspended colloids (e.g., complex proteinaceous polymeric molecular structures or clusters suspended in fluid) in nonliving systems, resulting in its kosmotropic character (see Table 4), which enables the salting-out known as “flocculation.” This useful tendency, for example in public water systems, can, however, be catastrophic in the blood and fluids of living organisms, where building blocks of necessary proteins are apt to be turned into useless debris linked to Al salts [59, p. 1410] and [60]. According to its Lewis acidity classification [61], Al^3+^ belongs in Class A, a small (hard) metal ion with low polarizability (deformability), preferentially forming ionic complexes with similar nonpolarizable ligands, particularly oxygen donors such as oxyanions of carbon, phosphorus, and sulfur—all of which are plentiful in living organisms—giving Al the potential to wreak havoc in living systems. For these reasons, Al is certainly not “inert,” nor is it biologically harmless [29–48]. As Table 1 shows, Al is causally linked to disorders in plants, animals, and humans [9, 28, 57], especially in the CNS of animals and humans.

Among the CNS problems in humans attributed to Al are dialysis associated encephalopathy (DAE) [32, 62], autism spectrum disorders [9, 63, 64], Alzheimer's disease, Parkinson's disease, and related dementias [28, 36] including those typical in Down syndrome [18]. Experimental and clinical data show the CNS as the most sensitive organ system negatively impacted by Al. Toxic effects manifest in impaired psychomotor control, altered behavior (i.e., confusion, anxiety, repetitive behaviors, sleep disturbances, deficits of speech, concentration, learning, and memory), and in potentially fatal seizures [18, 28, 38]. Al has been identified as the efficient cause of a whole class of immune dysfunctions directly involving the CNS and known as “autoimmune-inflammatory syndrome induced by adjuvants” (ASIA) [65–68]. As will be seen in this paper, the disorders with which Al has been associated as a causal factor are pervasive because they begin with the disruption of fluid structures involving water. Also, although Al negatively affects every layer of the body's biosemiotic systems, on which health depends, the symptoms of Al poisoning are often noticed when they inevitably reach and impact the CNS.

### 1.1. Aluminum in the Nervous System

As Table 2 shows, humans get about 95% of their Al burden from food [69] though estimates vary between 2 and 25 mg per day amounting to 14–175 mg per week [70–73]. In urban societies, the intake can exceed 100 mg per day, between 4 and 50 times the averages shown in Table 2. Because of increasing consumption of Al-containing convenience foods [74], in 2006, the Food and Agriculture World Health Organization Joint Expert Committee on Food Additives (FAO/WHO-JECFA) amended their provisional tolerable weekly intake (PTWI) for Al from 7 mg per kilogram of body weight (amounting to 490 mg per week for an average 70 kg human) to 1/7 of that amount. The Committee concluded that “aluminum compounds have the potential to affect the reproductive system and developing nervous system at doses lower than those” previously supposed [74]. Interpreting the averages in Table 2, using the estimated intake in urban settings as the higher end of the actual range, referring to the supposedly tolerable weekly intake based on the post-2006 numbers, average consumers weighing 70 kilograms are consuming between 2 to 100 times the provisionally estimated safe amounts of Al.

Given that severe toxic effects of Al occur in animal models at a concentration of 1.5 to 5 mg/kg of wet weight, independent of the mode of administration [75], it may be inferred that lethal poisoning of humans can occur at about 3–10 times the average amounts estimated to be absorbed by adult consumers studied. This leaves a narrow margin between the estimated average uptake and the lethal threshold of Al in the human CNS. Experiments on cats involved injecting Al into the brain and monitoring the response both behaviorally and physiologically [76]. Measured tissue levels of Al averaging 14 micrograms/gram were associated with extensive neurofibrillary tangles, which are a common feature of AD. This level is only marginally higher than the 9–11 micrograms/gram that have been detected in some regions of AD brains postmortem. This physiological effect was associated with observed impairment in short-term memory and acquisition of a conditioned avoidance response [77]. Al also causes a condensation of brain chromatin disrupting DNA transcription [78]. Animal models of neurological disease plainly suggest that the ubiquitous presence of Al in human beings implicates Al toxicants as causally involved in Lou Gehrig's disease (ALS) [44, 45], Alzheimer's disease [20, 21, 28] and autism spectrum disorders [9, 63].

### 1.2. The Toxic Effects of Aluminum as a Vaccine Adjuvant

Al salts (hydroxide and phosphate) are the most commonly used vaccine adjuvants and, until recently, the only adjuvants licensed for use in the USA [79–89]. In the absence of Al, according to their manufacturers, antigenic components of most vaccines (with the exception of live attenuated vaccines) fail to elicit the desired level of immune response [66, 80]. Although Al is neurotoxic, it is claimed by proponents that the concentrations at which Al is used in the vaccines do not represent a health hazard [19]. For that reason, vaccine trials often treat an Al adjuvant-containing injection as a harmless “placebo” (a comparison benchmark or control treatment) or they use another Al-containing vaccine to treat a “control group,” despite evidence that Al in vaccine-relevant exposures is universally toxic to humans and animals [9, 90, 91]. Its use in a supposed “placebo” or in any “control” treatment in vaccine trials is indefensible [95]. It is precisely analogous to comparing fire A against fire B, to make the argument that since A is no hotter than B, A is therefore not a fire.

During the last decade, studies on animal models and humans have shown that Al adjuvants by themselves cause autoimmune and inflammatory conditions [19, 79–81, 90, 95–103]. The animal models show that subcutaneous injections of Al hydroxide induced apoptotic neuronal death and decreased motor function in mice [2, 37–39] and sheep [43]. In newborn mice they were associated with weight increases, behavioral changes, and increased anxiety [2]. All these findings plausibly implicate Al adjuvants in pediatric vaccines as causal factors contributing to increased rates of autism spectrum disorders in countries where multiple doses are almost universally administered [9]. Also, as shown by Goldman and Miller in studies published in 2011 and 2012, strong correlations between infant mortality rates and the number of doses of vaccines administered also suggest deleterious impact of multiple exposures to their components [104, 105].

Follow-up experiments focusing on Al adjuvants in mice by Khan et al. [106] have shown that the adjuvants do not stay localized in the muscle tissue upon intramuscular injection. The particles can travel to the spleen and brain where they can be detected up to a year after the injection. Such findings refute the notion that adjuvant nanoparticles remain localized and act through a “depot effect.” On the contrary, the Al from vaccine adjuvants does cross the blood-brain and blood-cerebrospinal fluid barriers and incites deleterious immunoinflammatory responses in neural tissues [1–3, 9]. Tracking experiments in mice reveal that some Al hydroxide nanoparticles escape the injected muscle inside immune system cells such as macrophages, which travel to regional draining lymph nodes, where it can exit to the bloodstream gaining access to all organ systems, including the brain. As Khan et al. [106] have warned, repeated doses of Al hydroxide are “insidiously unsafe,” especially in closely spaced challenges presented to an infant or a person with damaged or immature blood brain or cerebrospinal fluid barriers [2]. Given macrophages acting as highly mobile “Trojan horses” [107], the Khan et al. warning suggests that cumulative Al from repeated doses in vaccines can produce the cognitive deficits associated with long-term encephalopathies and degenerative dementias in humans [40, 99].

The latest research by Luján et al. [43] described a severe neurodegenerative syndrome in commercial sheep linked to the repetitive inoculation of Al-containing vaccines. In particular, the “sheep adjuvant syndrome” mimics in many aspects human neurological diseases linked to Al adjuvants. Moreover, the outcomes in sheep were first identified following a mass-vaccination campaign against blue tongue and have now been successfully reproduced under experimental conditions following administration of Al-containing vaccines. Notably, the adverse chronic phase of this syndrome affects 50–70% of the treated flocks and up to 100% of the animals within a given flock. The disorder is made worse by cold weather conditions, suggesting synergy with other stress producing factors. The disorder is characterized by severe neurobehavioral outcomes—restlessness, compulsive wool biting, generalized weakness, muscle tremors, loss of response to stimuli, ataxia, tetraplegia, stupor, inflammatory lesions in the brain and the presence of Al in the CNS tissues, coma, and death [43]. These findings confirm and extend those of Khan et al. [106] who demonstrated the ability of Al adjuvants to cross the BBB, and they show that Al in the brain can trigger severe long-term neurological damage. The findings by Luján et al. [43] and Khan et al. [106] also show how and why reported adverse reactions following vaccinations are most commonly neurological and neuropsychiatric [6, 7].

### 1.3. Aluminum Disrupts Biosemiosis

The nervous system utterly depends on coherent signaling from the genome upward to psychological and social behaviors and is suited to induce entropy at these and the levels in between them. The long-term consequences involve many minute injuries, leading to inflammation, disorders, diseases, and the ultimate death of certain neuronal elements and possibly of the whole organism. As documented by Gryder et al. [108] in reference to cancer, disruptions in gene signaling and/or RNA transcription mechanisms induce a range of deleterious outcomes on protein formation. In turn, altered proteins impact cellular function. As Al moves in the body and CNA, it can create dysfunctional cells that foul signaling systems and neural circuits leading to additional dysfunctions and even behavioral aberrations. Immediately and cumulatively, Al-induced injuries tend to be expressed as abnormalities in the CNS trending toward ultimate fatality [109].

## 2. Biophysics of Aluminum Toxicity and Impact on Cellular Processes

The concepts of kosmotropic and chaotropic solutes (water structure makers and breakers), introduced by Collins and Washabaugh in 1985, have been used extensively by the biochemical and biophysical communities [110]. These concepts are highly relevant to this section. The reader is referred to Table 4 (above) for a summary of the concepts. According to Marcus (2012), when “the structural entropy according to [Barthel and] Krestov (1991) was compared by Collins (1997) to the entropy of pure water...for the alkali metal and halide ions, and Δ*S* = Δ_struc_ *S* − S* (H_2_O). Those with Δ*S* < 0 have large surface charge densities and are called kosmotropes (water structure making) whereas those with Δ*S* > 0 have small surface charge densities and are chaotropes (water structure breaking)” [111–113].

### 2.1. Al^3+^ Disrupts Water Dynamics of Biological Exclusion Zones

Al is a reactive element existing abundantly in nature but almost exclusively bound as mineral salts. Al salts are relatively insoluble except under acidic conditions, which are created by organic acids* in vivo* and adjacent to the exclusion zones (EZs) of biomembranes [114]. Concerning EZs, as argued by Ling [115] (also see his references), “under an ideal condition, an idealized checkerboard of alternatingly positively, and negatively charged sites of the correct size and distribution could polarize and orient deep layers of water molecules* ad infinitum*. Based on the quantitative data thus obtained and a relevant simple statistical mechanical law, the new theory predicts that a thin layer of water held between two juxtaposed ideals or near-ideal nanoprotoplasm (NP) surfaces will not freeze at any (attainable) temperature. On the other hand, water polarized and oriented by an ideal or near-ideal NP-NP system may also not evaporate at temperature hundreds of degrees higher than the normal boiling temperature of water” (p. 91). However, as Ling has also shown, Al has the power to alter these crucial EZs, disrupting their unique biophysical properties [116]. Or, as argued more recently by Davidson and colleagues, toxicants such as Al are invariably disposed to contribute to exogenous interfacial water stress (EIWS) in the critical EZs precipitating in vast numbers of minute toxic injuries, and leading to disorders, diseases, and sometimes catastrophic changes ending in fatalities [57, 59, 68, 117–119]. Concerning the many ways that toxicants in both their near and distant effects can increase biosemiotic entropy also (see arguments developed by Oller [17, 51], Gryder et al. [108], and Ho [52]). Shaw et al. (2013) have also presented data showing that biological water dynamics crucially enable quantum coherence across all biosemiotic systems [68].

### 2.2. Al^3+^ Speciation, Solubility, and Adsorption Are pH-Dependent

Conventional beliefs about Al safety [19] are rooted in the knowledge that, in the absence of citrate, insoluble Al compounds are poorly absorbed even if ingested [91]. However, the fact that Al hydroxide and phosphate solutions remain nearly saturated at neutral pH and standard temperature in pure water suggests that their poor solubility does not make them benign in living systems. Many other ligands besides water molecules can interact with Al when it is inhaled, ingested, topically absorbed, or parenterally injected. Acidic beverages such as soft drinks have a pH < 3; most fruit drinks have a pH < 4. Al in drinking water in concert with chemical agents that literally pull it out like claws—as suggested by the term* chelation*—can increase gastrointestinal absorption [107] and thus the biosemiotic entropy-inducing tendency of Al. Moreover, precipitates of Al need not be soluble to be toxic, especially in low pH compartments,* in vivo*, which favor more mobile hydrated Al^3+^ aqua ion, [Al(H_2_O)_6_]^3+^, as opposed to inner sphere contact ion pairs. According to Martin, the octahedral hexahydrate [Al(H_2_O)_6_]^3+^ dominates at pH < 5, and the tetrahedral [Al(OH)_4_]^−^ at pH > 6.2, while there is a mixture of species from 5 < pH < 6.2 [120, p. 12]. Adsorption and desorption of Al^3+^ species have long been known to demonstrate pH dependence [121, 122]. The aluminum aqua ion, [Al(H_2_O)_6_]^3+^, is well characterized in solution and the solid state [123]. In 1994, Marcus provided data indicating that, while [Al(H_2_O)_6_]^3+^ behaved like a typical strong kosmotrope, with a negative structural entropy value and enhancement of the H-bond structure of water, [Al(OH)_4_]^−^ demonstrated the properties of a chaotrope, with a positive structural entropy value and lessening of the H-bond structure of water [93]. Thus, it is clear from these data that pH has a major influence in determining the speciation, solubility, adsorption, and Hofmeister behavior [58, 59] of Al* in vivo*.

### 2.3. Glyphosate—A Ubiquitous Al^3+^ Chelating Agent

Being a modified form of glycine with both phosphonyl and carbonyl groups, glyphosate is already known to chelate metal cations [124]. Moreover, Al caged by glyphosate dimers and trimmers [125] bears a certain resemblance to chelation complexes of Al citrate. Given its biocidal effects on gut biota [126, 127], leading to inflammatory intestinal disorders commonly treated by Al-containing antacids [128], Al interacting with glyphosate is likely to increase its crossing of the endogenous intestinal biofilm barrier into the blood stream [129, 130]. Such Al-induced* leaking* of the endogenous biofilms of the gut and blood brain barrier could increase Al accumulation in the CNS. Glyphosate impairs the bioavailability of both tryptophan and methionine [126], and significantly reduced plasma concentrations of these amino acids have been found in Alzheimer's disease patients [131, 132].

Given the escalating use of glyphosate worldwide and the increasing incidence of inflammatory bowel disease [133] and gastroesophageal reflux disease [134], studies with animal models [135] are needed to assess the potential of glyphosate to specifically chelate and distribute Al compounds* in vivo*. High precision adsorption calorimetry may prove to be useful means of studying the thermodynamics of Al biosequestration, generally, and glyphosate Al chelation complexation,* in vitro* [136–138], specifically as suggested in Figure 2 from Guo and Friedman [139] which shows how Gadolinium (Gd^3+^) serves in biological cation sequestration. CNS delivery is known to occur, at least in part, via adsorptive transcytosis of cationized proteins and peptides [140]. This empiric observation, therefore, begs the questions: does glyphosate promote adsorptive transcytosis of Al, and* vice versa*; does Al promote adsorptive transcytosis of glyphosate, across the protective biofilms of the gut and blood brain barrier?

### 2.4. Al^3+^ Induces Oxidative, Genotoxic, and Interfacial Water Stress—A Triple Threat

A well-recognized effect of Al^3+^ is the induction of oxidative stress [141] and though it has prooxidant [142] effects through its impact on water dynamics as Ling has shown [143–145], it disrupts enzymes involved in the methylation pathway, increasing EIWS [59]. As a consequence, Al impacts epigenetic interactions and everything dependent upon them. As early as 1968, Riddick showed that Al^3+^ generally promotes agglomeration and precipitation even of anionic colloidal finely ground silica (minusil) [146]. Evidently, it does so in the same way that, in living organisms, Al^3+^ disrupts interfacial hydrogen bond (H-bond) cooperativity and the quantum coherence of water essential for cellular homeostasis.

### 2.5. Al^3+^ Disrupts H-Bond Cooperativity of Biological Water

The disruption induced by Al^**3+**^can be seen as a “red shift” of the stretching bands in the absorption spectra of water to longer wavelengths—thus a “bathochromic” shift—on both infrared and Raman spectroscopy. In 1985, Newton and Friedman employed a neutron diffraction method [147] to show that the dominant isotope effect of +3 ions is associated with the O–H stretch of the water. The shift to lower frequencies is proportional to the square of the ionic charge *z* in Na^+^, Mg^2+^, Al^3+^ (or, resp., 1, 4, and 9), while the oscillatory motion—the “libration” frequency—increases linearly with* z* in the same series (or, resp., 1, 2, and 3). More recent confirmation of this expectation has been produced in a series of papers by Probst and Hermansson (1992), Desiraju and Steiner (2001), Joseph and Jemmis (2007), and Jemmis and Parameswaran (2007) [148–151].

**Figure 1:**
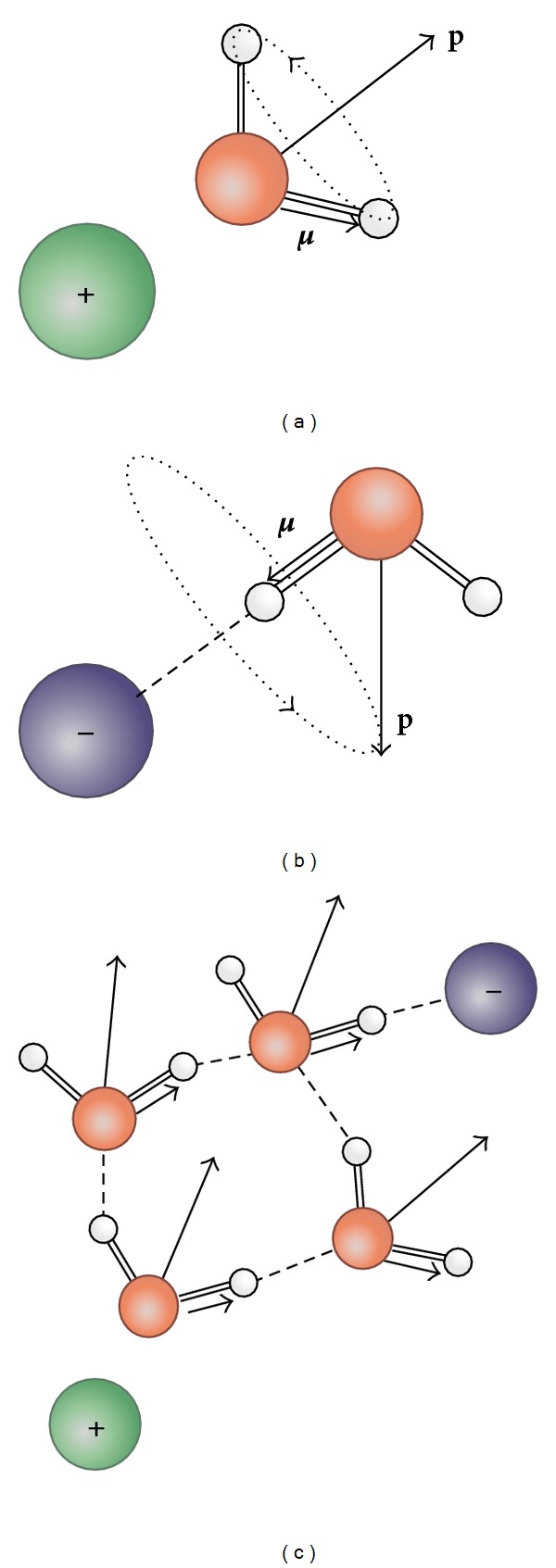
Semirigid hydration and cooperativity ((a) and (b)) a water molecule in the solvation shell of a cation (a) and an anion (b). Dielectric relaxation measurements probe the reorientation of the permanent dipole vector** p**. Femtosecond infrared spectroscopy is sensitive to the reorientation of the OD-stretch transition dipole moment ***μ***. The dotted arrows indicate reorientation in a cone, in the case of semirigid hydration. (c) Proposed geometry, in which the water dynamics are locked in two directions because of the cooperative interaction with the cation and the anion. Figure 1 is reproduced here from (Tielrooij et al. 2010) [153] with permission of the American Association for the Advancement of Science.

Light and electron microscopy also show that cell morphology is sensitive to EIWS [152]. Tielrooij et al. (2010) [153] employed both terahertz and femtosecond infrared spectroscopy showing that the effects of ions and counterions on water can be strongly interdependent and nonadditive, and, in certain cases, extend well beyond the first solvation shell of water molecules directly surrounding the ion [153]. They also found that, “if strongly hydrated cations and anions are combined, the dynamics of water molecules are affected, wherein* the hydrogen bond network is locked in multiple directions* (italics, ours)” as shown in Figure 1.

### 2.6. Al^3+^ Disrupts the Critical Metastable State of Neurolemmal Membranes

Al^3+^ dangerously shifts the intracellular balance that normally keeps macromolecules of DNA, RNA, and proteins from breaking up and disintegrating into an incoherent, disordered chaotropic mixture. This can lead to the disintegration of blood cells for example in hemolysis or, with equal harm, bioactive molecules combining in biologically useless ways into kosmotropic precipitates, forming dysfunctional molecular debris deposited on the walls of blood vessels (as in atherosclerosis, e.g.) or disabling neurons (as seen in the beta amyloid and/or hyperphosphorylated tau deposits characteristic of Alzheimer's plaques and tangles). To the extent that the membranous (plasmalemmal) material of all cells, along with the material linings of mitochondria, neurons, and neurofibrils, can be depolarized by Al^3+^; the loss of cytoskeletal conduction, much like an electrical circuit that “shorts-out” and burns, is certain to be injurious to macromolecules and to cells.

Some molecular damage can result in the orderly, and usually safe, disassembly of cells by apoptosis [154] or, with Al^3+^ toxicity, the disorderly disintegration which may release formerly contained pathogens and/or additional toxic debris, leading to necrosis and disease-enabling conditions. The noted effects of Al^3+^ can graduate from destroying macromolecules, plasmalemmal membranes, and whole cells to the destruction of tissues, organs, and even the death of the whole organism [155]. Studies on plant seedlings have shown an immediate effect on the cytoskeleton in which Al^3+^ causes a calcium channel blockade by its depolarization of membrane potential [156]. In both plants and animals, Al^3+^ blocks voltage-gated calcium channels and interferes with normal metabolism [157–162]. It also disrupts the stable water clusters found in highly structured multilayered EZs that serve as vehicles for storing incident radiant energy, as Chai et al. have shown [161].

Platt et al. (1993) demonstrated that extracellular pH modulates the Al blockade of mammalian voltage-activated calcium channel currents [163] at concentration range <200 *μ*M. Platt and Büsselberg (1994) then investigated the extracellular and intracellular effects of Al on voltage-activated calcium channel currents (VACCCs) in rat dorsal root ganglion neurons [164] and found that (a) Al applied extracellularly reduces VACCCs in a concentration-dependent manner, (b) the effect of Al was highly pH dependent in the investigated range (pH 6.4 to 7.8), and (c) there was evidence of intracellular as well as extracellular binding. They concluded that irreversibility, use dependence, and pH dependence, as well as binding sites for Al inside cells, contribute to its neurotoxicity. Platt and Busselberg also examined the combined actions of Pb^2+^, Zn^2+^, and Al^3+^ on VACCCs [164] showing that each of these metals reduced VACCCs, for all possible combinations, independent of the order of application. The impacts were additive and consistent with two metals acting at the same site as well as independent actions at different locations of the ion channel. Trombley (1998) demonstrated selective disruption of class A gamma-aminobutyric acid, the ligand gated ion channels (GABA_A_) receptors, by Al occurred with a minuscule concentration of <100 *μ*M in a culture of rat olfactory bulb neurons [165].

At the same time, and for some of the same reasons, ultrafast electron crystallography of' interfacial water by Pal and Zewail (2004) as followed by Oliveira et al. (2010) showed that recognition at the macromolecular levels of DNA, RNA, and protein is dependent on biological water dynamics in the 20–40 picosecond range [159, 160]. Based on the biosemiotic functions of such macromolecules, loss of such recognition would invariably lead to molecular mimicry, immune dysfunction, and the onset of autoimmune disease. Neuropathological states involving immune disorders can thus be conceptualized to arise from the breakdown of, or deviation from, the metastable critical state of biological water dynamics at the interphase of neuronal membranes. Similarly, with respect to neurological damage, Al has been shown to induce neuronal apoptosis* in vivo* as well as* in vitro* [166].

Sadiq et al. (2012) found that metal ions such as Al^3+^ tend invariably to target signaling pathways and may interact with various targets simultaneously. The long-range consequences show that ions interacting with any given molecular target can disrupt all of the processes dependent on it [162]. With respect to developmental neurological and other communication disorders, Oller and colleagues (2010a, 2014) have described this phenomenon as a domino or cascading effect [167–169] and Seneff et al. produced the same sort of argument for the biophysical level [57]. Likewise, Shaw et al. (2013) show how minimally stable states of interphase water at neurolemmal membranes can be upset by “noise” from Al^3+^ producing a “domino” effect [68] inducing long-wavelength perturbations leading to a cascade of energy dissipation on all scales [170].

### 2.7. Biological Water Modulates Biosemiotic Entropy at Multiple Levels Concurrently

Underlying all of the foregoing evidence, there is sound theory and a growing body of research (partially summed up in Figure 1) showing that water, rather than being a passive medium in which biological reactions take place, is an active participant [59, 60, 171]. With that in mind, it is plain that Al^3+^ must disrupt long-range, dynamical, interfacial H-bond cooperativity and that it must interfere with the quantum coherence of water, both of which are essential for cellular homeostasis. The geometry proposed by Tielrooij et al. (Figure 1), in which the water dynamics are locked in two directions, shows how the cation and anion produce the polarized-oriented multilayer (PML), confirming the theory of Ling (2003) [115], the exclusion zones (EZs) of water reported by Zheng and Pollack (2003) [114, 172, 173], and the H-bond cooperativity implicit in the EIWS theory [59]. Because of their chemical properties and affinities, Al^3+^ species tend to disrupt the hydrophobic surfaces of water based biofilms of all kinds. Al^3+^ disrupts such films by breaking down the complex hydrophobic forces binding the liquid. This kind of breakdown can be seen in its impact on the liquid films containing the peculiar colloids known as “coacervates” studied for the last 150 years by Lillie [174], Oparin and Synge [175], and numerous others, the recounting of which is found in Ling's work as cited. It also has the same disintegrative effect on the neurolemmal membranes throughout the body, showing how protoplasmic poisoning is invariably induced at many levels by the Al^3+^ species. The barriers between the blood and the brain and blood and the spinal cord, as well as the barriers protecting the blood and the rest of the body's tissues from the contents of the gut can be thought of as analogous to “exclusion zones” or differentiated “coherence domains” [172, 176], consisting in part or in whole of polarized-oriented multilayers of biological water as described by Ling [115] (and see his references).

Because of stretching and reorientation of H–O bonds, generalized from the dynamics illustrated in Figure 1, the local “unwetting,” “stretching,” and hydrophobic “collapse” of interfacial water can also disrupt signaling systems, leading to immune dysfunctions and autoimmune diseases, all beginning with EIWS [59, 68]. Also, for reasons already partially explained, the CNS is particularly susceptible to Al toxic damage, especially considering the critical role of biosulfates, both the HSPGs and, especially, the sulfoglycolipids such as sulfatide [57, 117, 118, 177] in the CNS. The latter are crucially involved in the formation of myelin, which is essential for healthy neural tissue and functions of the CNS and peripheral systems. Myelin, in turn, depends on HSPGs, which are essential in generating current and separating charge. But because myelin lipids and proteins demonstrate surface fractality over many scales [170, 178], toxic impact from Al and its compounds can do far-reaching harm. Also, it is known that Al^3+^, F^1−^, Hg^2+^, and Pb^2+^ are synergistically toxic and particularly so because of their affinity for biosulfates, such as the HSPGs.

**Figure 2:**
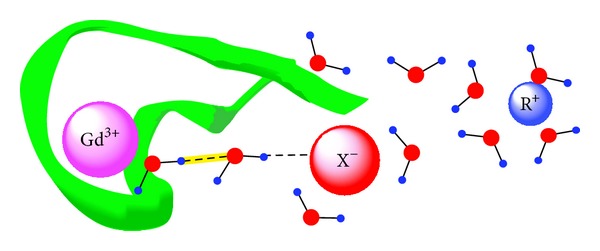
Depiction of how Gadolinium (Gd^3+^) vibronic side band luminescence spectroscopy (GVSBLS) acts as a probe of the coordination of biologically-relevant sites of cation sequestration. The figure is reproduced here from (Guo and Friedman 2009) [139] with permission of the American Chemical Society. Copyright 2009 American Chemical Society.

The anion in Figure 2 may be generalized conceptually to include the biosulfates, ROSO_3_^1−^ or SO_4_^2−^, fluoride (1^−^), carboxylates, oxyanions of nitrogen, and the biophosphates. The cation in this figure may also be generalized conceptually to include high charge density polycationic metals, such as Al^3+^, Hg^2+^, and Pb^2+^, as well as oxycations. If vectors (arrows with direction and magnitude) are employed, as in Figure 1 [153], the dynamical reorientation of the OD-stretch transition dipole moment vectors and permanent dipole vectors will result in polarization and orientation of multiple layers of water along the lines explained by Ling in 2003 [115].

### 2.8. Protoplasmic Poisoning via Cooperative Adsorption of Polycationic Metal Toxicants

In 2008, Harrison et al. found that certain heavy metal cations exert synergistic bactericidal and antibiofilm activity against* Pseudomonas aeruginosa* [179]. In May 28, 2008, Harrison et al. filed patent (U.S. 2008/0118573 A1) for use of heavy metals in the treatment of biofilms, including metal cations such as Mn^2+^, Co^2+^, Ni^2+^, Cu^2+^, Zn^2+^, Al^3+^, Ag^+^, Hg^2+^, Pb^2+^, Cd^2+^, Sn^4+^, and metalloid oxyanions. In 2010, Renslow et al. employed pulsed-field gradient nuclear magnetic resonance to study* in situ* effective diffusion coefficient (D_*rs*_) profiles in live biofilms [180] and observed distinctive spatial and temporal variation in D_*rs*_ for various locations in the biofilm. In 2013, Davidson et al. reviewed literature showing that, in several neurodegenerative and neuroimmune diseases, loss of anisotropy, loss of curvature, increase in diffusion magnitude, and loss of stiffness (softening), may be directly attributed to destructuring of interfacial water, which precedes overt signs and symptoms of oncologic, neurologic, and infectious disease [119, pp. 3851-3852].

Ling (1991) has argued as follows.

In autocooperative adsorption, the adsorption of an *i*th solute favors the adsorption of more* i*th solute; in a heterocooperative adsorption, the adsorption of an *i*th solute favors the adsorption of the alternative *j*th solute. Autocooperative behaviors, like those of a school of swimming fish and the sentinels guarding the Great Wall of China, tend to be all-or-none. … autocooperative adsorption is the backbone of coherent behavior in living cells including the maintenance of the living state [181, pp. 135–58].

Heterocooperative adsorption of Hg^2+^ solute would favor the adsorption of an alternative solute, such as Al^3+^ and vice versa, in a manner which tends to be all-or-none. Cumulative heterocooperative adsorption of cationic neurotoxicant metals, for example, Hg^2+^, Al^3+^, and Pb^2+^ explains their neurotoxic synergy and biosequestration.

### 2.9. EIWS Promotes Both Structural and Biosemiotic Entropy

The fact that Al^3+^ species are potent exogenous interfacial water stressors per the EIWS theory was elaborated by Davidson et al. [57, 59, 68, 117, 119, 177]; Marcus (2013) found, in his study of the incremental surface tensions of various elements, that Al^3+^ has one of the largest individual ionic surface tension increments (second only to La^3+^) [94]. This finding explains why Al^3+^ along with Hg^2+^ and Pb^2+^, as well as various cationic and nonionic surfactants, are potent factors in producing EIWS. Such observed facts explain how aluminum/phosphate and aluminum/sulfate species, either as the Al^3+^ aqua ion form at low pH or the inner sphere contact ion pairs at higher pH, by exceeding the incremental surface tension threshold of exclusion zones, can disrupt H-bond cooperativity [123]. In doing so they must augment biosemiotic entropy* in vivo*, tending toward dehydration as described by Sharma and Debenedetti (2012) [182].

In 1966 and 1967, Selye had already provided a comprehensive exposition of the toxicity of polyvalent metal ion salts [183, 184], particularly those with high charge density, leading to serial sensitization, resulting in both local and systemic thrombohemorrhagic phenomena, with microvascular ischemic and immune sequelae, in a highly stereotyped, pluricausal manner. The earliest events in the toxicity of Al^3+^ are biophysical, mediated by water, through disrupted interfacial H-bond cooperativity and quantum coherence [185–190]. Consistent with the red shift in Raman vibrational absorption frequencies discussed earlier and demonstrating it, in part, Falk (1984) had already found that a lowering of the bending frequency of water is associated with increasing cation charge and decreasing cation size [191]. Much more recently, Imoto et al. (2013) studied the origin of the difference in the H–O–H bend of the infrared spectra between liquid water and ice [192]. Furthermore, as suggested by Exley (2004) [142] and Mujika et al. (2011) [193], Al^3+^ may be predisposed to react* in vivo* with toxic impact on endogenous reactive oxygen species, such as the superoxide radical anion to form an Al-superoxide semireduced radical cation complex [AlO_2_^∙^]^2+^.

### 2.10. Distinctive Physical Properties of Al Species Determine Their Toxicity

Another unique property of Al ions is their high charge density. Ionic charge densities are reported in Table 3 using the methodology described by Rayner-Canham and Overton (2010) [194]. Also reported in the table are the crystal atomic radii as published by Shannon (1976) for the various ions [92]. The charge density of Al^3+^ is 372.6 C*·*mm^−1^ as compared to that of Gd^3+^ (91.5 C*·*mm^−1^), F^1−^ (16.2 C*·*mm^−1^), Na^+^ (24.5 C*·*mm^−1^), and Ca^2+^ (51.6 C*·*mm^−1^).

The high charge density of Al is a consequence of its relatively small radius and its fixed 3+ charge. These factors impact the solubility of the individual Al salts and their incremental impact on the surface tension of water [94, 195, 196]. With respect to biological impact, the vast array of enzymes and signaling proteins inhibited by Al species shows that Al toxicity is not limited merely to diffusion. The interaction of the various Al species with long-range, dynamical H-bond networks and the coherence domains of interfacial water suggests the involvement of nonthermal, magnetic [47], and quantum effects that are no doubt generalizable to many toxicants, particularly those with polycationic surfactants of high charge density (see Table 3).

Inorganic ions can be ranked on a chaotropic (disintegrative) to kosmotropic (colloid forming) gradient according to their enthalpy of hydration [197, 198] presented in Table 5 (above). The more negative the enthalpy of hydration, the more kosmotropic the solute. The opposite would indicate a chaotropic tendency. A formula that aids in understanding the relationship between charge density, radius, and enthalpy of hydration is given as follows:

(1) $$\begin{array}{c} H=-\frac{{Ze}^{2}}{2r}\left(1-\frac{1}{ℇ}\right), \end{array}$$

where *H* = Hydration enthalpy, *Ze* = Charge of the ion, *r* = Ionic radius, and *ℇ* = Dielectric constant of the solvent.

A smaller atomic radius and higher charge correlate with a more negative hydration enthalpy and greater kosmotropism—defined biologically as the tendency to cause macromolecular complexes in bodily fluids to form useless colloidal precipitates that are effectively sequestered from the water in organelles, cells, blood, lymph, protoplasm, or any bodily fluid. In biological systems, protein folding and unfolding (DNA also) depend on a delicate balance of chaotropic and kosmotropic forces on water [199]. Solutes sorted according to a chaotropic to kosmotropic gradient define the Hofmeister series [59]. In agreement with hydration enthalpies found in Table 5, Al^3+^ normally acting as a powerful kosmotrope plays havoc with the biological balance. In particular, the more kosmotropic a substance is, the more capable it is of salting-out proteins from an aqueous medium. Table 4 presents a comparison of the properties of chaotropic and kosmotropic ions.

The oxyphilic behavior of Al acting as a kosmotrope is shown in its avid binding to oxyanions of carbon, sulfur, and phosphorus [120]. Its lipophilicity, dose-dependence, time-dependence, and glial versus neuronal specificity have been studied by Campbell et al. (2001) [200] and as early as 1996, Bondy and Kirstein had already shown how Al species can promote iron-induced generation of harmful reactive oxygen species [201]. Cations such as Al can bind to *π* electrons within biomolecules [202]* in vivo,* inciting lipid peroxidation, DNA damage, and disruption of essentially all the biosemiotic systems deploying molecules containing calcium and sulfur [203]. A* prima facie* indicator of its toxicity is inflammation shown in cerebral markers elicited by chronic exposure to Al in drinking water [204]. Kiss (2013) has reviewed the coordination chemistry of Al^3+^ with small and large biomolecules, including serum components, and also the role of time in the distribution of this “sluggish” metal ion in a biological environment [205]. The results agreed with the computer model of Beardmore and Exley (2008), showing that Al has kosmotropic effects at a greater distance and more quickly than the “depot” theories could possibly explain [206].

The magnitude of the kosmotropic property of Al^3+^ can be seen in bold relief by comparing the degree of H-bond strengthening required to cause Al^3+^ to behave as a chaotrope [207]. If the H-bond energy of water increases, then various kosmotropic ions behave as chaotropes and vice versa. The required change in strength of H-bonds to cause Na^+^ to behave as a chaotrope is 11% strengthening and for K^+^ to behave as a kosmotrope is 11% weakening. The gradient between Na^+^ and K^+^ is almost two orders of magnitude smaller in comparison with the hydration enthalpy of Al^3+^ (−4690 kJ mol^−1^), in theory, the amount of energy released (as heat) when a mole of Al^3+^ dissolves into an infinitely diluted solution. The change of H-bond strength required for Al^3+^, a kosmotrope, to behave as a chaotrope is 1260.75% H-bond strengthening. The required H-bond strengthening is calculated by dividing the hydration enthalpy of the solute by the estimated isotropic point (−372 kJ mol^−1^). Table 5 shows selected hydration enthalpies of several common biologically relevant ions.

### 2.11. Molecular and Cellular Biosemiotic Disruption by Al^3+^ Is Concomitant

The foregoing facts and findings in this section help to show why and how Al^3+^ interacts synergistically with certain other toxic molecules and how it acts in producing or augmenting auto- and neuroimmune diseases. Kamalov et al. (2011) demonstrated the cytotoxicity on immune cells of environmentally common concentrations of Al (10–40 *μ*M) in murine thymocytes and lymphocytes [208]. Nearly all thymocytes showed evidence of damage at 30 *μ*M AlCl_3_ after only 5 minutes of incubation. A 60-minute exposure to 10 *μ*M AlCl_3_ caused damage of about 5% of thymocytes, while 50% were injured after 10 minutes at 20 *μ*M. In lymphocytes, injury was observed at 15 *μ*M AlCl3, and less than 50% of cells were injured after a 60-minute exposure to 20 *μ*M. Injury only rarely proceeded to rapid cell death and was associated with cell swelling. These results demonstrated a rapid dose-dependent injury in murine thymocytes and lymphocytes resulting from exposure to Al, as indicated by an increase in the entry into the cell of the DNA-binding dye, propidium iodide. The data suggest direct damage to the plasma membrane, manifested as an increase in membrane permeability, consistent with the EIWS theory.

Likewise, with respect to the synergistic interaction of Al^3+^ with Hg^2+^ species, Kern et al. (2013) examined the action of low levels, ≤1,000 nM, of thimerosal (49.55% Hg^2+^ by weight) on immortalized B-cells taken, respectively, from autism spectrum disorder subjects, their fraternal twins, a sibling, and an age/sex matched control. Observed contrasts showed impaired sulfation chemistry owed to the thimerosal exposure [209, 210]. In 2009, Pogue et al. presented data which underscores the potential of nanomolar concentrations of Al to drive genotoxic mechanisms characteristic of neurodegenerative disease processes [211, 212]. These data, combined with results reported earlier by Haley (2005), suggest toxic synergy between *μ*M Al^3+^ levels and nM thimerosal levels,* in vivo* [213].

While Al^3+^ can undoubtedly form complexes with proteins, nucleotides, nucleosides, RNAs, and DNAs, so too can stable nanoclusters of water, some of which are helical [214]. The presence of Al^3+^ could only create difficulties in such delicately balanced systems [215]. Also, given the growing body of empirical data suggesting that both gene structure and protein structure are dependent in part on interfacial water dynamics, it follows that the best known biological macromolecules depend in part on supramolecular systems [216, 217].

## 3. Corrupted Processes and Pathways Induced by Aluminum

### 3.1. Effect of Al on Iron Toxicity and Interference with BH4 and Calmodulin Function

Al is primarily transported in serum by transferrins [218]. Al may interact with transferrins at multiple candidate binding sites, including the transferrin receptors, thus influencing iron metabolism and transport. The fastest subunit of transferrins to react with iron is the tyrosinate complex [219]. Other amino acid residues with which Al may interact are aspartic acid, glutamic acid, and glutamine [220]. Al readily binds to apo-transferrin binding sites but does not compete with iron for binding with halo-transferrins. Al causes small conformational changes in transferrins without significant structural consequence [221], thus enabling transferrin receptors to actively transport Al across the blood brain barrier as if it were iron [222]. Once in the brain, displacement of iron from transferrins by Al results in iron toxicity and overproduction of reactive oxygen species by Fenton reactions [203, 223].

Six interactive cycles within the methylation pathway include (1) the urea cycle, (2) the tetrahydrobiopterin (BH4) cycle, (3) the folate cycle, (4) the methionine cycle (5) the S-Adenosyl methionine (SAM) cycle, and (6) the transsulfuration pathway. Dihydrobiopterin reductase (DHPR) is a critically important enzyme in the BH4 cycle that is inhibited by Al, and calmodulin (CaM) is critically inhibited in the urea cycle.

DHPR inhibition is implicated in Al induced encephalopathy [224]. Many accounts of Al toxicity are reported in the context of renal insufficiency. Al intoxication associated with pediatric renal insufficiency causes progressive encephalopathy in children [225]. Furthermore, Al intoxication by any cause such as occupational exposures will have the same inhibitory effect on DHPR [226]. BH4/BH2 ratios are decreased as a result of DHPR inactivation. BH4/BH2 ratios are reported to be decreased in Alzheimer's disease [28] and in autism [227]. About 60% of children on the autism spectrum are reported to experience clinical improvement after BH4 replacement therapy [228].

The folate cycle [229] enables components of urea, BH4, and methionine cycles to adapt to varying oxidative conditions. The dihydrofolate reductase (DHFR) system is a means of BH4 supply in cases of dysfunctional or inactive DHPR [230]. In this process, DHPR becomes more active in recycling BH4 from BH2 instead of acting on dihydrofolate to synthesize tetrahydrofolate when DHPR is functional. Congenital DHPR deficiency, such as in phenylketonuria (PKU) is associated with folate depletion [231] and treatment for PKU includes dietary folate replacement [232].

In addition, BH4 is cofactor for production of dopamine from tyrosine. Dopamine, cyanocobalamin, and 5-methyl tetrahydrofolate are required for synthesis of methionine from homocysteine [233, 234]. In Al toxicity, as in autism [63], dopamine becomes depleted because BH4 is depleted, further limiting remethylation of DNAs, RNAs, lipids, and proteins [235]. Furthermore, methionine is required to methylate DNA. The brain malformations seen in autopsies of autistic subjects [236] suggest failure of DNA methylation during brain development and growth.

In the urea cycle, BH4 is a cofactor with arginine in the synthesis of nitric oxide (NO) under endothelial nitric oxide synthase (eNOS). Not only does Al inhibit DHPR and production of BH4, but it also out-competes calcium for binding sites on calmodulin (CaM) causing conformational changes [237]. Properly bound with calcium, CaM is an essential cofactor in coupled eNOS mediated production of citrulline and NO from arginine. If BH4 is depleted or Al binds to calmodulin, eNOS follows an uncoupled pathway that favors production of peroxynitrite and superoxide. NO levels are paradoxically high in BH4 depletion, because it continues to be produced by alternate pathways, and its release from endothelial cells is inhibited by the high level of accumulated homocysteine [238].

High NO levels are associated with increased vascular permeability. NO stimulates mast cells and macrophages to release proinflammatory cytokines including IL-1, IL-6 tumor necrosis factor (TNF), and vascular endothelial growth factor (VEGF) [239]. This is the inflammatory profile found in autistic encephalopathy [240]. Accumulation of both reactive oxygen and nitrogen species results in severe oxidative and nitrosative stress [241–243].

### 3.2. Effects of Distinct Formulations of Aluminum Adjuvants: A Role for the Zeta Potential

As already noted, Al adjuvants are predominant modulators used in vaccines, although relatively little is known about how they work [244]. It was formerly claimed that Al adjuvants directly stimulate antigen-presenting cells by forming an antigen depot at the injection site [245]. Given the evidence that Al species used in adjuvants are readily transported throughout the body, the depot theory must be rejected. Others have proposed that Al stimulates dendritic cells, activates the immune complement system, and induces the release of chemokines [246]. It is generally agreed that Al hydroxide induces a Th2 type immune response [247, 248], whereas Al phosphate has been shown to induce a Th1 type response [249].

However, based on data from the CDC's Vaccine Adverse Event Reporting System (VAERS) database it is possible to compare the three distinct Al adjuvants used in the DTaP vaccine in particular (see Table 6): they consist of a hydroxide, a potassium sulfate, and a phosphate. The fact that all are used in the same multivalent vaccine minimizes the degree to which other factors, including the several antigens in the vaccine, might be influencing adverse reactions. Assuming only that all other factors excepting the Al adjuvants are held constant, an experimentally orthogonal comparison is possible among the three adjuvants. The method of comparison was a standard ratio of an expected value to the one obtained in each instance as susceptible to a standard chi-square distribution (the log-likelihood ratio) as described in [250].

The statistic in question expresses the likelihood that a given ratio of expected adverse reactions to actually observed adverse reactions could be attributed to chance. The critical probability for our tests was conservatively set at *p* < 0.05. The VAERS database for DTaP adverse reactions for the several formulations were compared with subsamples matched for age and number of cases. The comparison enabled the testing of experimental predictions concerning the relative mobility of charged particles in an electric field based on the Zeta potential (ZP) of the various Al adjuvants at issue. In blood—the most abundant fluid involved in transporting adjuvants from an injection site—the ZP reflects the negative charge of molecules attached to the membranes of suspended particles, such as red blood cells (RBCs) or lipid particles, which the Al^3+^ compound in any given case would be likely to link up with. A less negative ZP is associated with an increased tendency for RBCs to aggregate [251] that is, to form clots, whereas an even more negative ZP reduces that tendency.

The three DTaP formulations (Table 6) differ chemically only in their Al adjuvant component, as detailed by Caulfield et al. [244], and to that extent the vaccines differ in zeta potential (ZP). As those researchers found, ZP measured at pH 7.0 closely matching the value for blood, yielded a ZP value for hydroxide at +30 mV, for sulfate at 0 mV, and for phosphate at −20 mV: the sulfate formulation, therefore, should have the least impact. Using its ZP value at 0 mV as the baseline, it provided a reasonable estimate of the “expected value” for the ratio comparisons with the other two adjuvants to assess the impact of ZP on the adverse reactions reported. Results shown in Tables 7 and 8 show the outcomes for phosphate and hydroxide adjuvants. Compared to phosphate, local adverse events are reported more often for hydroxide, which, as expected, should migrate less from the injection site owing to a higher positive ZP, while phosphate should show the opposite effect owing to its negatively displaced ZP value.

The negative charge induces mobility owing to the electrical field induced by the voltage difference between arteries and veins [99, 100] while the positive charge tends to prevent mobility through the blood. The voltage difference is partly because the veins have a lower pH because CO_2_ is more acidic than O_2_. The lymphatic system, of course, as noted by Gherardi and colleagues [98–100], affords a bypass route that white blood cells (e.g., immune cells) can take (having penetrated the endothelial wall into the tissues) [252, 253]. However, this pathway also has the same voltage drop that would propel movement of negatively charged particles, as the lymph system returns to the venous system at the subclavian vein. On the other hand, positively charged particles would be stalled in the tissues as shown by Davidson and Seneff [59].

Thus, with the Al hydroxide adjuvant, we expect and find relatively more edema (swelling) at the injection site accompanied by “injection site reaction” and cellulitis because both plasma and lymphatic transit are stalled. Al phosphate, in contrast, with higher mobility and easier migration through the lymphatic system into the venous system, is more likely to reach distant areas including the brain, resulting, as observed, in a greater likelihood of systemic responses such as throat irritation, nausea, diarrhea, abdominal pain, and seizures. As expected, Al potassium sulfate did not produce any reactions with a *p* value under 0.05, when compared against either of the other formulations.

Observed syndromes associated with Al hydroxide include “macrophagic myofasciitis” (MMF) characterized by diffuse myalgia, chronic fatigue, and cognitive dysfunction, termed “mild cognitive impairment” [38, 40]. In a relevant study of that disorder, it was determined that the Al hydroxide adjuvant led to an accumulation of Al-loaded macrophages at the site of a previous intramuscular immunization [39]. Given the results reported in Table 7, it must be inferred that macrophages lingering at the injection site on account of the elevated ZP associated with the hydroxide formulation are responsible for this observed syndrome. Likewise, the autoimmune syndrome recently identified by Shoenfeld and colleagues [65–67] is consistent with the generalized toxicity of the Al adjuvants.

### 3.3. Aluminum Interactions with Fluorine

Fluorine is the most chemically reactive nonmetal and the most electronegative element [254]. According to Martin (1996) [255], Al^3+^ binds F^−^ more strongly than 60 other metal ions tested. Even with micromolar concentrations of Al^3+^, these two atoms react to form AlF_4_^−^, a molecule whose shape and physical properties closely resemble those of the phosphate anion, PO_4_^−2^. This feature has been exploited to help researchers understand phosphate-dependent reactions in signaling cascades [255–258]. For example, it has been shown, by exploiting AlF_4_^−^, that melatonin's widespread signaling effects are mediated by G-proteins [259]. However, if AlF_4_^−^ forms whenever these two elements are both present, it is known to interfere with regulatory GTP hydrolases which play an initiating role in phosphate-based signaling cascades [260, 261]. Should the AlF_4_^−^ mimetic, which is not responsive to the GTPase, stick in the “on” position, an overresponsive cascade of transcription, motility, and contractility, as well as apoptosis would proliferate. If this were to happen, such interference, for which Al toxicity affords many alternative routes remaining to be explored, is certain in all cases to augment biosemiotic entropy.

Strunecká and Patocka proposed that the toxic role of Al in Alzheimer's disease may be predominantly due to the formation of AlF_4_^−^ [262]. The formation of that complex, according to experimental evidence, in quantities as little as 1 ppm of fluoride contamination of water supplied to rats led to greater uptake of Al into the kidney and brain along with the formation of amyloid deposits like those in Alzheimer's disease [263]. As proteins, RNAs, and DNAs become damaged through oxidation [264–267], if they cannot be repaired, failure of the lysosomal and mitochondrial organelles will lead to apoptosis [268–270] or, in worse cases, to necrosis. Al compounds can only contribute to such outcomes in a negative way.

Prior research has also shown that insufficient sulfate in the extracellular matrix of all the tissues, particularly the endothelial wall, plays a significant role in disorders and disease conditions [59, 117, 177, 199]. Heparan sulfate populates the glycocalyx in the capillaries [118, 271–273] and enables a low-resistance capillary wall permitting smooth blood flow [57, 59, 68, 117, 177, 199]. Sphingosine-1-phosphate-induced Rac activation, chemotaxis, and angiogenesis associated with endothelial cell migration are mediated by G-proteins [274].

With all of the above considered it may be notable that postmortem examination of Alzheimer's brains reveals severe deficiency in sulfatide, a myelin-specific sulfated sphingolipid, which normally makes up 6% of the lipid content and is especially concentrated in the myelin sheath [275]. Twenty-two subjects in the early stage of Alzheimer's disease showed a depletion of 93% in gray matter and up to 58% in white matter in all brain regions examined. Aside from an overabundance of ceramide, the precursor to sulfatide (ceramide was elevated threefold in white matter), all other lipid parameters appeared normal. This outcome was not associated with a defect in sulfatide synthesis, so the pathology appears to involve breakdown of sulfatide to provide sulfate to the vasculature, critical for maintenance of an adequate supply of oxygen and nutrients to the brain.

**Figure 3:**
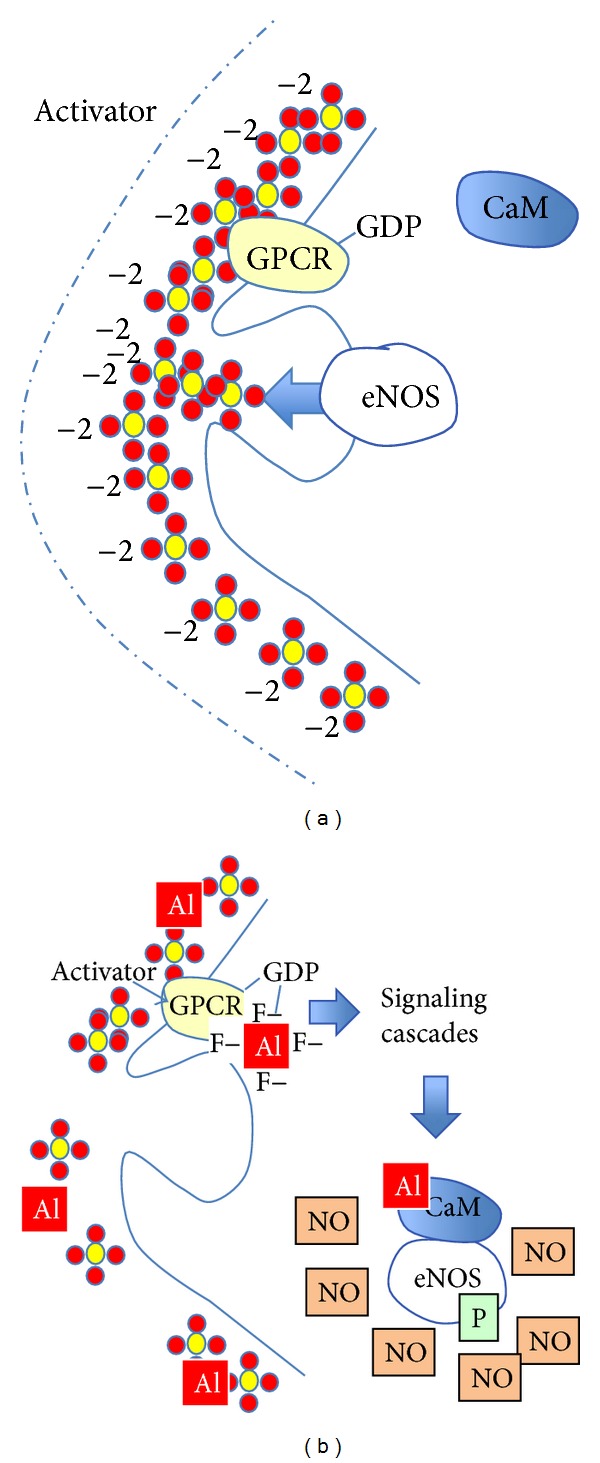
Illustration of the devastating effects of Aluminum on a typical cell related to sulfate inactivation, G-protein signaling, and calmodulin signaling. (a) A healthy cell without Al contamination. eNOS, attached to the membrane at a caveola, produces sulfate, which maintains a healthy glycocalyx with sufficient negative charge. (b) Al binds to the sulfates, eliminating the negative charge, which allows cytokines to penetrate through the glycocalyx, activating G-protein coupled receptor signaling cascades. AlF_4_^−^ disrupts the signal, acting as a phosphate mimic, and Al binds to CaM, inducing eNOS detachment from the membrane. Phosphorylation cascades activate eNOS to produce abundant NO released into the cytoplasm, instead of producing sulfate to enrich the glycocalyx.

Seneff et al. previously suggested that endothelial nitric oxide synthase (eNOS), an enzyme present in endothelial cells, RBCs, and platelets, among other cell types, is a “moonlighting” enzyme, which synthesizes sulfate when it is attached to caveolin in the plasma membrane and synthesizes NO (which is converted to nitrate within a few seconds) when it is phosphorylated and bound by a calcium-CaM complex in the cytoplasm [118]. These findings suggest that eNOS plays the dual-purpose of regulating the balance between kosmotropes and chaotropes in the cytoplasm of the cell and also enabling the proper folding and functions of cellular proteins [199], as detailed in Figure 3.

## 4. Discussion

Considering all the ways Al^3+^ is known to impact biological systems negatively, as summed up in Table 1, exposure to that cation generally disrupts biosemiotic cascades. Its effects lead to minute cumulative injuries to DNAs, RNAs, cellular proteins, and lipids through glycation and oxidation damage, as well as impaired lysosomal recycling of debris, and, ultimately, in some cases, leads to cell death by necrosis. Death by apoptosis, the preferred alternative, may also follow Al-induced injuries and changes in DNAs, RNAs, proteins, and any downstream mediators. For example, MMF has been shown to manifest with Al retention at the injection site of vaccines containing Al hydroxide [38, 39] and far-reaching negative effects on the body's immune systems can be seen in ASIA owed to eventual migration of Al adjuvants away from the injection site [65–67]. Given its positive differential impact on ZP, Al hydroxide has been shown to linger at the injection site for many months, although it eventually is transported into brain by macrophages [77]. In that particular case, the normal apoptosis of injured cells is disrupted by the high electrostatic attraction of the Al^3+^ ion towards the negatively charged sulfates in the glycocalyx actually forcing the Al^3+^ cation to penetrate and traverse the viscous water of the exclusion zone. The result is disruption of the structured water in the exclusion zone, compromising the glycocalyx barrier and allowing signaling molecules to gain access and launch a G-protein mediated cascade reaction.

This cascade is intensified by the effects of AlF_*x*_ on G-protein signaling, and the subsequent disruption of cellular metabolism follows. When the cell becomes necrotic, having skipped over any possibility of normally regulated and orderly apoptosis, it virtually disintegrates, releasing DNA and other cellular debris into the interstitial spaces to degenerate or to be carried away by the lymphatic system. In the case of the other less confined Al adjuvants that can more readily migrate away from the injection site, the confusion induced in biosemiotic systems is the predictable source of a confused and self-destructive autoimmune response as seen in ASIA. The downstream result is an immune attack on cells, tissues, and organs throughout the body but especially in the CNS, as seen in diseases such as multiple sclerosis and other demyelinating conditions.

It is clear that Al^3+^ toxicity, interacting synergistically with other toxicants such as solvated species of Hg^2+^, Pb^2+^, F^−^, AlF_*x*_ (aluminofluoride), SiF_*x*_ (silicofluoride), glyphosate, and including chelation complexes, must directly increase biosemiotic entropy on multiple levels simultaneously by disrupting long-range, dynamical, interfacial hydrogen bond cooperativity and the quantum coherence of water. The outcome is widespread (systemic) and involves virtually simultaneous inhibition of many different enzyme systems. It is therefore unsurprising that Al^3+^ is associated with anaphylaxis and sudden death [59]. The data from the studies reviewed here show that the complex coacervate protoplasm, studied now for about 150 years [145, 174, 175], is susceptible to poisoning by high charge density polyvalent cations, for example, Al^3+^, Hg^2+^, and Pb^2+^. Empirical studies [93, 94] of ion solvation suggest that local order induction can result in loss of long-range, systemic coherence and cooperativity [185]. On a supramolecular biosemiotic level, EIWS induced by Al^3+^ disrupts interfacial hydrogen bond cooperativity and quantum coherence of biointerfacial water. At a critical threshold, the self-ordered criticality of biointerfacial water collapses. The most notable effects of this sort occur in the CNS [68, 276].

In the larger context, however, Al toxicants can themselves, or by synergistically interacting with other toxicants, destroy cells in any organ system, although none are more vulnerable than the CNS and the peripheral systems attached to it. While significant everywhere in the body, the impact of biosemiotic entropy in the CNS is critical because of the nested and highly interdependent systems connected to it. For example, the loss of neural cells (neurons or glia) in the CNS tends to disrupt circuits that depend on such cells. In turn, groups of neurons in functional nuclei can be rendered dysfunctional through the loss of individual neuronal elements. In the same way, the loss of functional nuclei can lead to catastrophic stress on the CNS itself and/or on dependent organ systems. Fatality may be preceded by a cascading series of failures resembling the collapse of complex interdependent networks [277].

An additional factor that makes the nervous system uniquely vulnerable is the highly specified differentiation of neuronal activities due to sequenced developmental programs. These programs, acting in response to both genetic and environmental instructions, ensure that the loss of functional circuits cannot be easily replaced, since the very milieu into which they might be integrated (e.g., stem cells) differs from one stage of development to the next during which some window, or “critical period,” for neuroplasticity may have passed. While it is true that critical periods vary between neuronal regions (human association versus primary cortex, e.g.), younger nervous systems appear to have a greater capacity for recovery following injuries to organ systems provided stem cells remain intact. However, damage to the DNA of stem cells is apt to be irreparable even in early stages of development, and Al^3+^ can cause both injuries to organs and DNA damage directly impacting stem cells.

A third reason for the notable toxicity of Al^3+^ is that neurogenesis—that is, the birth of new neurons—is relatively rare in the adult CNS in most regions. Compared to the ability of other organs to regenerate, for example, the skin or liver, the CNS has limited capacity to do so, which renders it more vulnerable to irreversible damage at fairly early stages of development. Thus, Al and its compounds have remarkable power to harm neurons and to produce systemic damage. The observed impact may, in some instances, be sudden, as in anaphylaxis and sudden death syndrome, but in other instances, it may build slowly to a crisis level through chronic doses leading to systemic autoimmune responses as in the vaccine-induced disorders. The variable range of toxic effects in ASIA, for example, can best be explained in terms of the biodistribution and pharmacokinetics of the particular Al adjuvant used. Some of the observed differences depend predictably on ZP and its impact on interfacial water tension.

**Figure 4:**
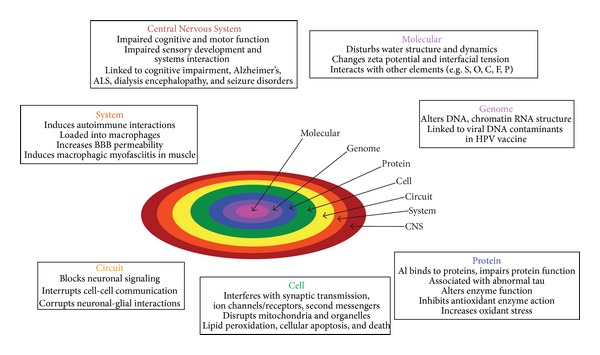
Schematic of the biosemiotic levels at which Al can impact the body and CNS.

Figure 4 is a two-dimensional schematic showing some of the ways Al and its compounds can impact the biosemiotic systems encompassed by the CNS. The summary suggests a nested biosemiotic hierarchy of ranked systems communicating within and across levels. In ascending order, they range from molecules to genes, proteins, cells, circuits, CNS subsystems, and the CNS itself. Impacts at any level can induce changes in those above and below them. For example, Al actions at a cellular level will necessarily perturb protein structures and DNA (the levels below) and alter cell-to-cell communication at the circuit level (above). Although Figure 4 focuses on the deleterious effects of Al on the nervous system, it should be clear that its impacts are systemic.

## 5. Conclusions

Aluminum induces entropy in living organisms by disrupting all levels of structure from water molecules through all biosemiotic systems. Entropy-inducing cascades, feedback loops (positive and negative) within and across levels, can damage DNAs, RNAs, proteins, cells, tissues, and whole organ systems. As a result of cellular damage caused by an Al compound, injured and dying cells will release proteases, excitatory amino acids, and ions (e.g., potassium, calcium), disrupting biosemiosis at many levels. Toxic effects of Al and its compounds thus tend to proliferate. Interactive results involving immune functions, for instance, make the impact worse than if only one system were involved. Of course, the dose-response of Al and its compounds must be considered, but even at low doses, especially with repeated exposures, Al can have cumulative deleterious effects that can be extreme and even fatal. For that reason, a repeated low dose exposure may prove more damaging than a single larger dose. Al and its compounds can cross biosemiotic levels, damaging genetic systems, proteins, cells, and all systems up through the CNS. While higher doses may rapidly affect multiple levels, as in dialysis-associated encephalopathy (DAE), low doses over time, for example, from vaccines, can degrade metabolism and disrupt repair and defense systems and can spiral out of control as in ASIA. Al adjuvants in vaccines may hyperdrive the immune functions of the body but they also directly disrupt biosemiotic systems. Sound theory, empirical research, and reasonable inferences from sources cited here show that Al and its compounds damage biological systems. Such conclusions warrant considerations at a policy level to limit human exposure to Al and its compounds.

## Figures and Tables

**Table 1:** Common sources of Al compounds and their immunoneurotoxicological effects in humans and animals.

Aluminum source/compound	Dose & duration	Route	Species	Adverse effects
Standard infant feeding solution	~20 *μ*g/kg/day, >10 days	Intravenous (parenteral)	Human, premature infants	Reduced developmental attainment at the corrected post-term age of 18 months, as evidenced by significantly lower Bayley Mental Development Index (BMDI) scores (mean loss of one point on the BMDI/day of full intravenous feeding, after adjustment for potentially confounding factors) compared to infants fed with Al-depleted solutions [31].

Al-containing dialysis fluid (derived from Al-sulfate treated tap water)	1 ppm, chronic (2–5 years)	Intravenous	Human, dialysis patients (15–61 years old at the start of the dialysis treatment)	Speech impairments (stuttering, dysarthria, dyspraxia, and motor aphasia), movement disorders (twitches, tremors, myoclonic jerks, seizures, and motor apraxia), cognitive impairments and behavioural changes (progressive dementia, paranoia, confusion, and psychosis), and death [32].

Al-containing antacids	Chronic	Oral	Human infants	Craniosynostosis (premature ossification of the skull and obliteration of the sutures) [33].

Various dietary	Chronic	Oral	Elderly human subjects	Impaired visuomotor coordination, poor long-term memory, and increased sensitivity to flicker (correlated with high Al-serum levels) [34].

Al sulfate (present as flocculant in potable water supplies, accidentally released in high amounts)	500–3000 x the acceptable limit under European Union Legislation (0.200 mg/L), chronic (15 years)	Oral	Human adult (female, 44 years old)	Sporadic early-onset *β* amyloid angiopathy (Alzheimer's-related disease), difficulty in finding words, progressive dementia, visual hallucinations, headache, anxiety, cerebral ischemia, and death [35].

Al-containing food pellets	0.5–1.7 mg/kg/day (typical human), chronic (22–32 months)	Oral	Rats, 6 months old at the start of treatment	Cognitive deterioration and impaired performance in learning tasks, impaired concentration, and behavioral changes including confusion and repetitive behaviour [36].

Al lactate	500–1000 ppm, chronic (during gestation and lactation)	Oral	Mice dams	Hind limb paralysis, seizures, and death (dams), lower neurobehavioral development and altered performance on a neurobehavioral test battery in pups (foot splay, forelimb, and hind limb grip strengths reduced) [37].

Al hydroxide as a vaccine adjuvant	1–17 doses of Al-containing vaccines (hepatitis B, hepatitis A, and tetanus toxoid) in the period of 10 years prior to disease diagnosis	Intramuscular injection	Human adult macrophagic myofasciitis (MMF) syndrome patients (mean age 45 years)	MMF typical clinical manifestations: myalgia, arthralgia, chronic fatigue (disabling fatigue >6 months), muscle weakness and cognitive dysfunction (overt cognitive alterations affecting memory, and attention manifested in 51% of cases) [38–41]. Typical histopathology: presence of granulomatous myopathological lesion comprised of Al-hydroxide-loaded macrophages at the site of vaccine injection (usually deltoid muscle); persistence of Al long-term, up to 8–10 years in postinjection mice [38, 39, 42]. 15–20% MMF patients concurrently develop an autoimmune disease, most frequently being multiple sclerosis-like demyelinating disorders, Hashimoto's thyroiditis, and diffuse dysimmune neuromuscular diseases (i.e., dermatomyositis, necrotizing autoimmune myopathy, myasthenia gravis, and inclusion body myositis); even in the absence of overt autoimmune disease, low titres of autoantibodies, increased inflammatory biomarkers, and abnormal iron status commonly detected in exposed mice [38, 39].

Al hydroxide as a vaccine adjuvant	14 injections over a 6-month period	Subcutaneous	Sheep, male 3 month old lambs	“Sheep adjuvant syndrome” first identified following mass-vaccination for bluetongue; experimentally reproduced by repetitive injection with Al-containing vaccines [14]; observed in acute form (affecting 25% of exposed flocks, 0.5% animals within a flock) and chronic phase form (affecting 50–70% of all exposed flocks and up to 100% of animals within a given flock). Acute phase symptoms: lethargy, reluctance to move, bruxism (teeth grinding), transient blindness, nystagmus (rapid abnormal eye movements), stupor, abnormal behavior, disorientation, and a low response to external stimuli, seizures, and occasionally death; histopathological lesions mainly consisting of acute meningoencephalitis (similar to those observed in humans postvaccination) and demyelinating foci Chronic phase symptoms: severe neurobehavioral outcomes including restlessness, compulsive wool biting, generalized weakness, muscle tremors, loss of response to stimuli, ataxia, tetraplegia (paralysis of all four limbs), stupor, coma, and death. Inflammatory lesions (multifocal neuronal necrosis and neuron loss in both dorsal and ventral column of the gray matter) and presence of Al in CNS tissues [43].

Al hydroxide as a vaccine adjuvant	2 injections, 2 weeks apart	Subcutaneous injection (behind the neck)	Mice, 3 months old CD-1 male	Motor neuron degeneration and apoptosis, motor function deficits, decrease in strength, cognitive deficits, and decreased performance in learning tasks, decrements in spatial memory, activation of microglia [44, 45].

Al oxide fumes, occupational exposure	0.13–1.95 mg/m^3^, chronic	Inhalation	Human, adults (mean age 39 years)	Headache, emotional irritability, concentration difficulty, insomnia, mood lability [46].

**Table 2:** Estimates of daily and weekly intakes of Al in humans [28, 74].

Major sources of Al exposure in humans	Daily Al intake (mg/day)	Weekly Al intake (mg/day)	÷PTWI^†^ (1 mg/kg/bw; for an average 70 kg human PTWI = 70 mg)	Amount delivered daily into systemic circulation (at 0.25% absorption rate)
Natural food	1–10 [2, 8, 23–26]	7–70	0.1–1	2.5–25 *μ*g
Food with Al additives	1–20 (individual intake can exceed 100) [3, 5, 18]	7–140 (700)	0.1–2 (10)	2.5–50 *μ*g (250 *μ*g)
Water	0.08–0.224 [2, 8, 21]	0.56–1.56	0.008–0.02	0.2–0.56 *μ*g
Pharmaceuticals (antacids, buffered analgesics, antiulceratives, and antidiarrheal drugs)	126–5000 [1, 2, 8]	882–35,000	12.6–500	315–12,500 *μ*g
Vaccines (HepB, Hib, Td, DTP)	0.51–4.56 [9]	NA	NA	510-4560 *μ*g^‡^
Cosmetics, skin-care products, and antiperspirants^§^	70 [1, 9]	490	NA	8.4 *μ*g (at 0.012% absorption rate) [10, 11]
Cooking utensils and food packaging	0–2 [2]	0–14	0–0.2	0–5 *μ*g

^†^PTWI (provisional tolerable weekly intake) is based on orally ingested Al, generally only 0.1–0.4% of Al is absorbed from the GI tract, however, Al may form complexes with citrate, fluoride, carbohydrates, phosphates, and dietary acids (malic, oxalic, tartaric, succinic, aspartic, and glutamic), which may increase its GI absorption (0.5–5% [70, 82]). Coexposure to acidic beverages (lemon juice, tomato juice, and coffee) also increases Al absorption as well as conditions of Ca^2+^, Mg^2+^, Cu^2+^, and Zn^2+^ deficiency [70, 83–85].

^‡^A single dose of vaccine delivers the equivalent of 204–1284 mg orally ingested Al (0.51−5.56 mg), all of which is absorbed into systemic circulation [86, 91]. Al hydroxide, a common vaccine adjuvant has been linked to a host of neurodegenerative diseases; it also induces hyperphosphorylation of MAP tau *in vivo* [44, 45, 87].

^§^The risk of antiperspirants is both from dermal exposure and inhalation of acrosols. Al is absorbed from the nasal epithelia into olfactory nerves and distributed directly into the brain [88, 91].

**Table 3:** A comparison of the physical properties of metallic Al with those of its common competitors in biological systems [89]. Crystal ionic radius source: [92]. Magnetic susceptibilities source: [47, pp. 4-131 to 4-136]. Viscosity *B* coefficient source: [93]. Standard molar electrostriction volume source [94].

	Mg	Al	Ca	Mn	Fe	Co	Zn
Atomic number	12	13	20	24	25	27	30
Electron configuration	[Ne]3s^2^	[Ne]3s^2^3p^1^	[Ar]4s^2^	[Ar]4s^2^3d^5^	[Ar]4s^2^3d^6^	[Ar]4s^2^3d^7^	[Ar]4s^2^3d^10^
Ionization energies (kJ/mol)	737.7 1450.7 [7732]	577.5 1816.7 2744.8 [11577]	589.8 1145.4 [4912.4]	717.3 1509 [3248]	762.6 1561.9 [2957]	760.4 1648 [3232]	906.4 1733.3 [3833]
Crystal ionic radius (pm)	86	67.5	114	97	92	135	88
Electron affinity (kJ/mol)	0	42.5	2.37	0	15.7	63.7	0
Electronegativity (eV)	1.31	1.61	1.0	1.55	1.83	1.88	1.65
Magnetic susceptibility (*X*_*m*_/10^−6^ cm^3^ mol^−1^)	+13.1	+16.5	+40	+511	*Ferro-magnetic *	*Ferro magnetic *	−9.15
Charge density (coulombs*·*mm^−1^)	120.1	372.6	51.6	143.7	98.1	154.9	112.1
Viscosity *B* Coefficient (dm^3^ mol^−1^, 298.15 K)	0.385	0.75	0.289	0.390	0.42	0.376	0.361
Standard molar electrostriction volume (−Δ_elstr_ *V*_*i*_)/(cm^3^ mol^−1^)	52.5	59.3	38.5	30.7	—	38.5	—

**Table 4:** Summary comparisons of chaotropic versus kosmotropic ions.

Chaotropes (water-structure breakers)	Kosmotropes (water-structure makers)
Typically larger radius, singly charged ions with low charge density	Typically small radius, often multiply charged ions with high charge density
Interact more weakly with waters than water molecules interact with each other	Interact more strongly with waters than water molecules interact with each other
Interfere little with hydrogen bonds of the surrounding waters	Capable of weakening and breaking hydrogen bonds of the surrounding waters
Decrease surface tension	Increase surface tension
Reduce viscosity	Increase viscosity
Increase nonpolar solubility	Decrease nonpolar solubility
Unfold proteins	Stabilize proteins
Destabilize hydrophobic aggregates	Stabilize hydrophobic aggregates and bonding
Increase solubility of hydrophobic solutes	Reduce solubility of hydrophobic solutes
Salt in proteins	Salt out proteins
Net positive entropy of ion solvation	Net negative entropy of ion solvation

**Table 5:** Selected hydration enthalpies of common biologically relevant ions [89].

Symbol	Δ*H*_hydr_ (kJ mol^−1^)	Source
NO_3_^−^	−312	[198]
K^+^	−321	[197]
NH_4_^+^	−329	[198]
HSO_4_^−^	−368	[198]
Cl^−^	−371	[197]
HCO_3_^−^	−384	[198]
Na^+^	−413	[197]
OH^−^	−520	[198]
H_2_PO_4_^−^	−522	[198]
SO_4_^2−^	−1035	[198]
H^+^	−1100	[197]
Ca^2+^	−1650	[197]
Mg^2+^	−1920	[197]
Mg^2+^	−1949	[198]
Al^3+^	−4690	[197]

**Table 6:** Three different formulations of the DTaP vaccine and the number of reported adverse reactions available from VAERS for each one.

Formulation	Adjuvant	Adverse reactions
Tripedia	Aluminum potassium sulfate	11,178
Daptacel	Aluminum phosphate	8,786
Infanrix	Aluminum hydroxide	13,238

**Table 7:** Adverse reactions reported in VAERS for sulfate versus hydroxide in age-matched samples, and the likelihood that the contrasts observed in these distributions could have occurred by chance (*p* < 0.05).

Condition	Sulfate	Hydroxide	*p* value
Swelling	2210	2665	0.0066
Cellulitis	445	617	0.020
Pain	622	815	0.020
Fever	2032	2296	0.034
Injection site reaction	393	520	0.038
Injection site swelling	7	33	0.045

**Table 8:** Counts of various adverse reactions reported in VAERS for sulfate versus phosphate in age-matched equal subsets of the sample space, and the likelihood that the contrasts observed in these distributions could have occurred by chance according to a log likelihood ratio test. Included are all the reactions for which phosphate was more common with a *p* value under 0.05.

Condition	Sulfate	Phosphate	*p* value
Hospitalization	177	363	0.0044
Seizures	186	333	0.011
Rotavirus	3	47	0.013
Abdominal pain	6	53	0.014
Nausea	203	338	0.015
Diarrhea	95	174	0.028
Pneumonia	13	50	0.032
Dehydration	12	48	0.032
Throat irritation	81	147	0.036

## Highlights

- Aluminum (Al^3+^), suspected as a toxicant for 100 years, injures the CNS and immune systems, individually and synergistically.
- Al^3+^ disrupts biological water dynamics and macromolecules: DNAs, RNAs, proteoglycans, and proteins.
- Al^3+^ disrupts H-bond cooperativity interfering with the quantum coherence of living systems.
- Al^3+^ interferes with biological signaling—biosemiosis—from the very lowest to the highest levels in the nervous system.
- The effects are synergistic with other toxicants, including mercury, lead, fluoride, and glyphosate.
